# The Effect of Core Stabilization Exercises on Upper Extremity Function and Balance in Children with Cerebral Palsy: A Randomized Controlled Trial

**DOI:** 10.3390/healthcare13121454

**Published:** 2025-06-17

**Authors:** Kübra Ecem Küçük, Cigdem Cekmece

**Affiliations:** 1 Department of Physical Therapy and Rehabilitation, Kocaeli University, Umuttepe Campus, Izmit 41001, Turkey; 225317001@kocaeli.edu.tr; 2 Section of Occupational Therapy, Department of Therapy and Rehabilitation, Vocational School of Kocaeli Health Services, Kocaeli University, Umuttepe Campus, Izmit 41380, Turkey

**Keywords:** cerebral palsy, core stabilization exercises, upper extremity functions, balance

## Abstract

**Aim:** This study explores the effects of core stabilization exercises on balance and upper extremity functions—both unilateral and bilateral—in children with cerebral palsy (CP). **Method:** Thirty-six children with CP (aged 5–12) were randomly assigned to a study group (*n* = 18) or control group (*n* = 18). Both groups received four weeks of physiotherapy and occupational therapy. The study group additionally performed ~45 min of daily core stabilization exercises. Outcome measures included the Box and Block Test (BBT), Assisting Hand Assessment (AHA), Pediatric Berg Balance Scale (PBBS), and Trunk Control Measurement Scale (TCMS). (ClinicalTrials.gov ID: NCT06973213). **Results:** No significant baseline differences were found between the groups (*p* > 0.05). Post-intervention, the study group showed significant improvements in PBBS (*p* = 0.011), TCMS static sitting (*p* = 0.003), dynamic reaching (*p* = 0.037), and total score (*p* = 0.044). Between-group differences remained non-significant for BBT, AHA, and TCMS selective movement control (*p* > 0.05). Within-group analysis revealed significant gains in BBT (median = 7), PBBS (median = 8), TCMS total (median = 12), static sitting (median = 3.5), and selective movement (median = 6) (all *p* < 0.001). AHA showed a near-significant trend (median = 6, *p* = 0.051). **Conclusions:** Core stabilization exercises significantly enhance balance and unilateral upper extremity function in children with CP. However, they show limited impact on bimanual function. Integration of these exercises into rehabilitation programs may optimize motor outcomes.

## 1. Introduction

Cerebral palsy (CP) refers to a group of disorders that impact movement and posture, resulting in activity limitations. Motor problems such as muscle weakness, spasticity, lack of selective motor control, and balance disorders hinder voluntary movement in children with CP [1]. Additionally, these impairments can cause difficulties in independent standing, walking, upper extremity use, and other activities of daily living (ADLs) [2,3].

Trunk control is responsible for the proper and coordinated function of the upper and lower extremities, facilitating the performance of ADLs [4]. Core stability can be considered a crucial component of trunk control. The core region is essential not only for maintaining movement and balance but also for optimizing the kinetic chain functions of the upper and lower extremities. A stronger and more stable core contributes to the more effective use of the extremities [5].

Children with diplegic CP typically exhibit impaired mediolateral stability, whereas those with hemiplegic CP show reduced anteroposterior stability [6], affecting not only sitting and standing but also reaching and walking [7]. Additionally, weakness and poor coordination in the postural muscles contribute to limitations in upper extremity function, especially in tasks requiring precision and motor control [1,8,9].

Approximately 60% of children with CP aged 4–14 experience more than mild hand function difficulties (MACS > I). While 87% of children with spastic unilateral CP achieve age-appropriate manual independence (MACS I–II), this rate drops to 63% in bilateral CP and only 20% in dyskinetic CP [10]. These upper extremity challenges negatively affect essential functions such as play, grasping, reaching, and object manipulation [11,12]. Moreover, deficits in core stability are directly linked to reduced upper limb performance, with improved trunk control shown to enhance the functional use of the hands [4].

Balance is a complex motor, sensory, and cognitive function that involves the adjustment of posture during functional activities through sensory inputs from the proprioceptive, vestibular, and visual systems [13]. Studies on balance have found that children with CP exhibit weaker static and dynamic balance reactions compared with typically developing children [13,14].

Research has shown that core stabilization exercises not only improve functional use of the upper extremities but also enhance dynamic sitting and standing balance, trunk control, and gait [15,16]. However, most existing studies have primarily focused on the relationship between core stabilization and either balance or gait [17,18,19,20]. While the association between core stability deficits and upper extremity limitations is well documented, recent studies have begun exploring targeted interventions to address these challenges. Core stabilization programs designed to enhance trunk control and proximal stability have emerged as promising approaches to improving motor outcomes in this population. Prior randomized controlled trials have demonstrated the effectiveness of such programs in improving unilateral upper extremity function, dynamic balance, and gait. For example, Abd-Elfattah et al. [15] reported significant gains in hand dexterity following a core exercise program in children with hemiplegic CP. Similarly, El Shemy [16] observed improvements in trunk muscle endurance and gait. However, these studies primarily focused on unilateral upper extremity performance or isolated balance outcomes, leaving a gap in the understanding of how core stabilization may affect bimanual coordination—an essential factor in daily functional independence.

The number of randomized controlled trials investigating the effects of core stabilization exercises on upper extremity function in children with CP remains limited [15,21,22]. Moreover, existing studies have assessed upper extremity function only unilaterally. To date, no studies have examined bimanual hand use, despite its critical role in daily life participation. In the study by Akbaş & Günel [21], improvements in both upper and lower extremity function were reported following core stabilization exercises; however, as with prior studies, only unilateral hand function was evaluated.

Improving both balance and upper extremity function is crucial for enhancing the daily independence and participation of children with CP. While many rehabilitation approaches focus on isolated domains (e.g., gait or hand function), few interventions simultaneously address trunk control, balance, and upper limb use. Core stabilization exercises offer a clinically promising strategy to improve multiple functional domains at once, potentially enhancing real-world outcomes such as dressing, self-care, and play. Thus, this study addresses an important clinical gap by evaluating the combined effects of these exercises on balance and both unilateral and bimanual hand use.

## 2. Materials and Methods

This study was conducted as a randomized (using closed envelopes and simple randomization method), single-blind, controlled clinical trial with a parallel design at the Department of Physical Medicine and Rehabilitation, Kocaeli University, -Izmit/Kocaeli, Turkey- between March 2024 and December 2024. The children were divided into study and control groups, each consisting of 18 participants, using simple randomization with the closed-envelope method. All parents/caregivers of the children with CP participating in the study were informed about the study, and written informed consent was obtained from the parents/caregivers before the children participated. This study was approved by the Kocaeli University Ethics Committee (KAEK/01.b1.02). The study was registered in Clinical Trials Registry (NCT06973213) on 7 May 2025.

The trial was completed as planned, reaching both the target sample size (*n* = 36) and predefined endpoint (December 2024) without early termination. No stopping rules were triggered, as confirmed by our independent data-monitoring committee.

Before starting the study, a power analysis was performed using the G*Power 3.1.9.4 program with α = 0.05 and Power (1 − β) = 0.80. The outcome measure was the percentage change in the two groups based on the initial measurement of the PBBS test, which was one of our primary outcomes. Using the obtained results (means and standard deviations) from this study, we calculated the effect size as 1.04 and a total sample size of 32 (*n*1 = 16, *n*2 = 16). Anticipating potential data loss, the study was conducted with 36 participants (*n*1 = 18, *n*2 = 18).

The study included patients aged 5–12 years diagnosed with CP and followed up at the Department of Physical Medicine and Rehabilitation, Kocaeli University, who had no other medical conditions that could affect the evaluation and had not had surgery in the past six months. Children with severe intellectual disability, muscle contractures or bone deformities, visual impairments, uncontrolled seizures, other neuromuscular diseases, spinocerebellar ataxia, autism spectrum disorder, or communication disorders, as well as children diagnosed with hyperactivity and attention deficit disorder, were excluded from the evaluation. Patients classified as level IV or V according to the Gross Motor Function Classification System (GMFCS), with a spasticity level of 3 or higher according to the Modified Ashworth Scale (MAS) and classified as level IV or V according to the Manual Ability Classification System (MACS), were not included in the study.

To ensure allocation concealment, an independent, central allocation protocol was used in our study, ensuring that no individual associated with the study was involved in the allocation process. To maintain standardization, all assessment measurements were performed by a blinded, independent evaluator who was unaware of the patient allocation.

The sociodemographic information of the children with CP participating in the study was recorded, including their gender, age, and CP type. The GMFCS was utilized to evaluate gross motor function levels, MACS to assess the levels of manual ability, and MAS to measure spasticity levels. To measure the children’s upper extremity bimanual functions, the Assisting Hand Assessment (AHA) was used, and for unilateral functions, the Box and Block Test (BBT) was applied. The Pediatric Berg Balance Scale (PBBS) and the Trunk Control Measurement Scale (TCMS) were used to assess balance. No additional analyses beyond the protocol-specified methods were conducted.

This study had no missing data; all enrolled participants (*n* = 36) provided complete datasets for all timepoints. No interim analyses were planned or conducted in this study. No predefined stopping guidelines were established (e.g., for serious adverse events or clear demonstration of efficacy). The trial was designed to continue until the predetermined sample size was reached.

Evaluations took place in a calm, well-lit, ventilated room with a comfortable temperature, ensuring a safe and pleasant environment for the children. Various materials including balls, mats, chairs, parallel bars, and steps were utilized for the PBBS and TCMS assessments. For the AHA evaluation, performed by a certified and trained therapist, foot-supporting steps, as well as appropriately sized tables and chairs, were provided. The therapist was positioned directly in front of the children during the assessments. An environment was set up with age-appropriate toys from the AHA kit, enabling children to use both hands while performing all upper extremity functions. A camera was placed at an appropriate angle behind the therapist, and a 15 min recording was made for each child. The video footage was then viewed, scored, and recorded.

### 2.1. Randomization Procedure

Participants were randomized to either the control or study group via computerized simple randomization using the RAND () function in Microsoft Excel, which generated a sequence of ‘1’ (study) or ‘2’ (control) codes. To ensure allocation concealment, the codes were placed in sequentially numbered, light-proof, opaque envelopes, each labeled with a participant number and group allocation. The envelopes were sealed and prepared by an independent research coordinator uninvolved in recruitment or assessment. After enrollment, participants randomly selected the next consecutively numbered envelope (rather than drawing from a pool) to maintain sequence concealment. The administering physiotherapist opened the envelope to reveal the assignment, while assessors (therapists) and the statistician remained blinded (single-blind design). Envelope integrity was monitored throughout the trial, and the allocation sequence was inaccessible to study personnel until intervention assignment. Outcome assessors and data analysts were blinded to group allocation. Participant and intervention provider blinding was not feasible due to the nature of the exercise therapies. Blinding was attained by (1) using different intervention and assessment personnel, (2) evaluation standardization protocols, and (3) coded datasets. Envelope integrity and allocation concealment were both maintained during the trial.

### 2.2. Assessment Scales

#### 2.2.1. Assisting Hand Assessment (AHA)

AHA measures how effectively the assisting hand is used to perform bimanual tasks. Since ADLs typically require the use of both hands, bimanual performance is crucial when considering hand function. AHA assessments are observational and sensitive to change. The bimanual activities scored in the test were semi-structured according to age, allowing interaction with the physiotherapist. The results obtained from AHA assessments were used to guide interventions and measure changes over time [23].

The test involves two stages: Initially, the evaluator (such as a physiotherapist) sits directly in front of the child and arranges an environment that enables the child to perform all upper extremity functions using both hands by offering age-appropriate toys from the AHA kit. A video recording of approximately 15 min is made with the camera positioned at an appropriate angle behind the physiotherapist in advance. After the recording, the physiotherapist reviews the footage and scores each activity on a scale of 1 to 4 (4: effective use; 3: partially effective use; 2: ineffective use; 1: inability to use).

#### 2.2.2. Box and Block Test (BBT)

The BBT is used to assess gross grasp and the speed of hand dexterity in children. It consists of 150 small (2.5 cm) wooden cubes placed in a wooden box divided into two compartments by a partition. The child is instructed to transfer the cubes as quickly as possible from the compartment corresponding to the tested hand to the adjacent empty compartment. The child is asked to transfer one cube at a time. The test is explained to the child, and a 15 s practice trial is given. Then, the number of cubes transferred within 60 s is recorded. The test is administered to both the affected and unaffected hands [24].

#### 2.2.3. Pediatric Berg Balance Scale (PBBS)

The PBBS is a modified version of the original Berg Balance Scale designed to assess balance in children, particularly those with motor impairments or developmental disabilities. It consists of 14 tasks that measure balance in various contexts, such as sitting, standing, reaching, turning, and transitioning between positions. The PBBS helps evaluate a child’s ability to maintain and control their balance, providing important information for clinical decision making and treatment planning. Each item is scored on a scale from 0 to 4, and the maximum total score is 56. A Turkish validity and reliability study for children with CP was conducted in 2002 [25].

#### 2.2.4. The Trunk Control Measurement Scale (TCMS)

The Trunk Control Measurement Scale (TCMS) is an assessment tool used to evaluate trunk control. The TCMS assesses static trunk control during sitting balance, wherein the upper and lower extremities remain fixed, and static trunk control during extremity movements. Dynamic trunk control during sitting is divided into two categories: selective motor control and dynamic reaching. The scale consists of 15 items in total. The TCMS total score ranges from 0 to 58, with higher scores indicating better trunk control. For the TCMS, static sitting balance, selective motor control, dynamic sitting balance, dynamic reaching, and the total score are calculated. The Turkish validity and reliability of this scale were established through a study conducted by Ozal et al. [26].

### 2.3. Intervention

All children with CP who participated in the study underwent a physiotherapy and rehabilitation program, including passive/active-assistive/active range-of-motion exercises, strengthening exercises, passive/active stretching exercises, weight-bearing exercises, approximations, protective reaction training, balance exercises, balance exercises on unstable surfaces, standing exercises, single-leg stance training, proprioceptive training, ambulation exercises inside and outside the parallel bars, stair climbing and descending, in-place jumping, forward jumping, step jumping, and dual activities involving both the upper and lower extremities. The intervention was conducted for 4 weeks, 5 days a week, with each session lasting 45 min.

As part of occupational therapy, all children participated in reaching activities, weight-shifting exercises, sensory training, and activities targeting hand–eye coordination. Additionally, bimanual activities such as stringing beads, tearing paper, cutting paper with scissors, stacking Legos, striking two plates together, throwing and catching a ball, opening a bottle cap, clapping, opening a pencil case, and playing with playdough were included. Furthermore, exercises focusing on gross grasp (e.g., picking up balls of various sizes, cone-shaped objects, and blocks) and fine grasp (e.g., handling coins, keys, screws, and paper) were implemented. The intervention was conducted for 4 weeks, 5 days a week, with each session lasting 45 min.

Children included in the study group received core stabilization exercises in addition to the traditional physiotherapy program and occupational therapy for a duration of 4 weeks, 5 days per week, with each session lasting 45 min. Core stabilization exercises were performed on a soft mat under the supervision of the same physiotherapist. To prevent the risk of falls and injuries, children were placed on the mat, and any potentially hazardous objects in the surrounding area were removed. Throughout the sessions, the physiotherapist remained next to the children at all times. No sharp, cutting, or skin-irritating materials were used in the toys and equipment involved in the therapy. Both interventions were delivered per protocol by trained physiotherapists. Adherence was > 90% in both groups, with fidelity ensured via video audits (intervention) and checklists (control) (Table 1).

In this study, core stabilization exercises were applied in a fixed and standardized order to ensure strict adherence to the intervention protocol and enhance comparability across participants. The decision not to randomize the exercise sequence was made to minimize potential variability that could influence the intervention outcomes and to maintain procedural consistency.

The intervention began with breathing exercises. The core exercises included pelvic bridging on stable and unstable (with and without a ball) surfaces, sit-ups, double knee-to-chest exercises, cat-camel exercise, clam exercise, supine twist exercise with a ball between the knees, mini squats, donkey kicks and side donkey kicks, seated trunk rotations with cross-reaching, reaching, and perturbation exercises. Each exercise was performed in two sets of 10 repetitions, with a one-minute rest between sets. The number of repetitions for each exercise was adjusted according to the child’s tolerance and performance.

Children with CP were treated with age-appropriate intervention design. All activities were designed with a game-based methodology to capture the interest of children and maximize participation. For instance, exercises in balance were mixed with tasks like “obstacle courses” and “target ball-throwing”. Core stabilization exercises included the use of colored balls, foam cubes, and interactive playthings. Bimanual activities (e.g., Lego assembly, bead threading) were included in daily play activities. The treatment was modified according to the developmental and cognitive needs of young children. For example, “bridge building” activities were combined with “tunnel crawling” activities. Animated images and music were used to maintain interest. These adaptations made the program engaging, appropriate to the developmental level of young children, and effective. Sessions were split into 15–20 min slots to coincide with children’s concentration levels, with physical/playful breaks in between. For example, a single 45 min period was broken up into 3 × 15 min modules (exercise + play + free activity). Low-level incentives like stickers and oral praise were provided after every module to maintain encouragement. Exercises were tailored according to GMFCS and MACS levels. For example, supported sitting positions were used for diplegic CP children, and standing activities were designed for hemiplegic CP children. For monitoring adherence and fatigue, physiotherapists recorded the attendance and performance of every child on a daily basis using logs. Simplified exercise charts were given to the caregivers for practicing at home for consistency. With all this, for inattention and fatigue management, physiotherapists tracked behavioral signs (yawning, restlessness, loss of concentration) and dynamically controlled the activity intensity. For fatigued children, session length was reduced or breaks lengthened.

Adverse events were defined as any exercise-related discomfort (e.g., pain, fatigue, or musculoskeletal injury) leading to modification or discontinuation of the protocol. They were systematically assessed using a standardized form completed by therapists at each session, recording pain intensity (VAS), fatigue, and other symptoms. Participants/parents could also report events spontaneously. No serious adverse events were observed.

#### Integration of the F-Words Framework in Our Intervention

We structured the treatment applied to children with CP within the framework of the ICF’s (International Classification of Functioning, Disability and Health) F-words (Functioning, Family, Fitness, Fun, Friends, Future) model. The six F-words framework reorients attention in pediatric disability toward essential dimensions of child development. These principles, grounded in the ICF, translate theoretical concepts into practical, everyday strategies for children with disabilities and their families, reflecting the core elements of a child’s holistic well-being [27]. The implementation of these six F-words in our treatment was as follows:

Functioning: The intervention prioritized functional goals through practical tasks such as reaching, grasping, and postural control exercises (e.g., Pediatric Berg Balance Scale, Trunk Control Measurement Scale). These aimed to enhance engagement in daily routines, reflecting the ICF’s emphasis on activity and participation.

Family: Parental collaboration was central to the program, with caregivers participating in therapy sessions and home practice. Families received tailored strategies to integrate exercises into daily life, ensuring consistent support. Parents also contributed to setting personalized objectives aligned with their children’s needs.

Fitness: The program emphasized active motor tasks to boost energy and stamina, blending play with structured exercises. Dynamic activities targeting gross and fine motor skills were prioritized, with sessions structured to balance exertion and recovery to sustain engagement.

Fun: Playful methods, including imaginative scenarios, narrative-driven games, and movement challenges, were employed to maintain enthusiasm. Activities were customized to individual preferences, fostering enjoyment and self-driven participation in therapy.

Friends: Peer engagement was encouraged through collaborative exercises and group tasks. The therapeutic environment emphasized inclusivity, fostering shared experiences and mutual encouragement among participants.

Future: While the study did not address long-term transitional planning (e.g., adolescent readiness), this limitation underscores the need for future research to explore sustained outcomes.

### 2.4. Statistical Analysis

Statistical analysis was performed using IBM SPSS 29.0 (IBM Corp., Armonk, NY, USA) software. To determine the sample size of the study, G*Power version 3.1.9.7 (Kiel University, Kiel, Germany) software was used. The normality of distribution was assessed using the Shapiro–Wilk Test. Numerical variables were presented as median (25th–75th percentile) and frequency (percentages). Differences between groups for non-normally distributed numerical variables were compared using the Mann–Whitney U Test. Differences between measurements for continuous variables that were not normally distributed were evaluated using the Wilcoxon signed-rank test. To assess differences between groups for categorical variables, Fisher’s Exact Chi-square test, Yates’ Chi-square test, and Monte Carlo Chi-square test were used. A *p*-value of < 0.05 was considered statistically significant for two-tailed tests.

## 3. Results

**Figure 1:**
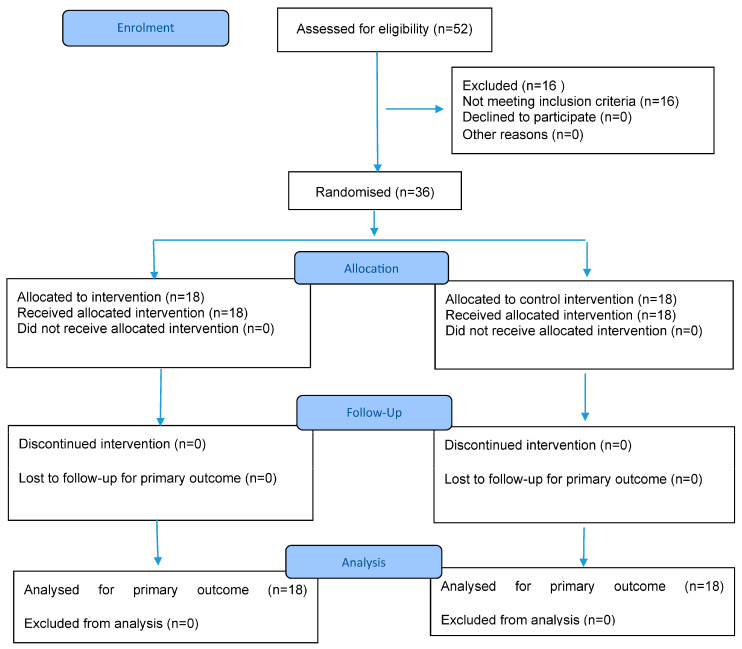
CONSORT 2025 Flow Diagram. CONSORT flow diagram template courtesy of https://www.consort-spirit.org (accessed on 1 June 2025).

The study was conducted at the Department of Physical Medicine and Rehabilitation, Faculty of Medicine, Kocaeli University, between March and December 2024. A total of 52 children with spastic CP were initially screened and assessed for age, gender, CP type, and inclusion and exclusion criteria. Sixteen children with CP were excluded from the study as they did not meet the inclusion criteria (nine patients were at GMFCS levels IV and V, two patients had mental retardation, and five patients were under the age of 5). A total of 36 children (study group *n*: 18, control group *n*: 18) who met the selection criteria were included in the study (Figure 1).

This study consists of 36 children with spastic CP. The children were randomly divided into two groups: the study group (18 children, 9 girls and 9 boys) and the control group (18 children, 8 girls and 10 boys). The median age of the study group was 5.5 years, while the median age of the control group was 7.5 years. The study group consisted of 6 children with hemiplegic type and 12 children with diplegic type CP, while the control group consisted of 3 children with hemiplegic type and 15 children with diplegic type CP. According to the GMFCS levels, an equal number of patients were found in levels I, II, and III in the study group. In the control group, there were 4 children in level I, 5 in level II, and 9 in level III. According to the MACS levels, 8 children in the study group were at level I, 7 children at level II, and 3 children at level III, while in the control group, 8 children were at level I, 4 children at level II, and 6 children at level III. No significant differences were found between the two groups in terms of demographic characteristics (*p* < 0.05). Demographic information of the patients is provided in Table 2.

A statistically significant correlation was found both between BBT and PBBS (*p* = 0.014, r = 0.406) and TCMS/static sitting balance (*p* = 0.084, r = 0.292), TCMS/selective movement control (*p* = 0.006, r = 0.448), TCMS/dynamic reaching (*p* = 0.018, r = 0.392), TCMS Total (*p* = 0.005, r = 0.453); and AHA and PBBS (*p* = 0.013, r = 0.410) and TCMS/selective movement control (*p* = 0.010, r = 0.425), TCMS/dynamic reaching (*p* = 0.029, r = 0.364), TCMS Total (*p* = 0.011, r = 0.418). The correlation between upper extremity unilateral/bilateral functions (BBT and AHA) and balance parameters (PBBS and TCMS) in all patients with CP is presented in Table 3.

Before treatment, there was no significant difference between the groups in the parameters of BBT, AHA, PBBS, and TCMS (*p* > 0.05). After treatment, significant improvements were observed in both groups in intragroup comparisons of BBT, AHA, PBBS, and TCMS parameters compared with pre-treatment results (*p* < 0.05). However, in the control group, no statistically significant difference was observed in the intragroup comparison of the dynamic reaching subtest of TCMS (*p* > 0.05). Although within-group improvements were observed in BBT, AHA, TCMS/selective movement control but no statistically significant difference was found between the study and control groups post-treatment (*p* > 0.05). However, significant differences were observed in favor of the study group in the other parameters (PBBS, TCMS/Static sitting, TCMS/Dynamic reaching, and TCMS Total) (*p* < 0.05). (Table 4 and Table 5). Additionally, between-group comparison of difference scores of the study and control groups for BBT, AHA, PBBS, and TCMS were analyzed statistically, and significant differences were found in favor of the study group for all parameters, except for the AHA evaluation parameter (*p* < 0.001) (Table 6).

Additionally, Cohen’s d effect sizes were calculated for all statistically significant outcomes, and except for the BBT parameter, all other assessment measures (including PBBS, TCMS subscales, and AHA) showed larger effect sizes in the study group compared with the control group (Table 4, Table 5 and Table 6).

The analyses were strictly limited to the pre-specified investigations outlined in the original study protocol. No post hoc analyses were performed based on any unexpected findings during data collection.

## 4. Discussion

This randomized controlled trial aimed to evaluate the effects of core stabilization exercises on the upper extremity function and balance in children with CP. To our knowledge, this is the first study to concurrently assess the impact of core stabilization on both unilateral and bimanual upper limb function, along with balance, in this population. While previous randomized controlled trials have explored these outcomes individually, the interrelationship between upper limb function, balance, and bimanual coordination—and the role of core stability within this dynamic—has not been thoroughly investigated. By addressing all three domains together, this study offers a more integrated perspective on the synergistic role of core stabilization in enhancing motor function in children with CP. Throughout the study, participant safety and adherence were high. No adverse events occurred, and no issues such as distress, fatigue, or emotional withdrawal were observed during treatment sessions.

Our study assessed unilateral upper extremity motor functions using the BBT, bimanual motor functions with the AHA, functional balance with the PBBS, and trunk control with the TCMS. The results obtained from this randomized controlled study indicate that, except for the dynamic reaching subtest of TCMS in the control group, improvements were observed in all parameters in both groups. When analyzing the differences between groups, significant improvements were found in favor of the study group in PBBS, TCMS/Static sitting, TCMS/Dynamic reaching, and TCMS total parameters, whereas no significant changes were observed in the other parameters. However, in the between-group comparison of difference scores of the study and control groups, statistically significant improvements were observed in favor of the study group for BBT, PBBS, and all TCMS parameters. In contrast, no significant difference was found in AHA scores.

In this study, no significant difference was found between the groups in BBT scores at the end of the four-week treatment period. However, when comparing the difference scores between groups, a significant improvement was observed in favor of the study group. This suggests that core stabilization exercises may enhance upper extremity function by improving postural control and proximal stability. Nonetheless, the absence of a significant post-treatment difference limits attributing this improvement solely to the intervention, indicating that other contributing factors may also be involved. For instance, internal and external sensory inputs—key elements in the rehabilitation of individuals with CP—may have played a role. Furthermore, it has been proposed that core stabilization enhances proximal stability, which is essential for effective distal mobility and postural control mechanisms [15]. While our findings support a potential association between core stabilization and upper extremity mobility, further research is needed to determine whether these improvements are clinically meaningful and sustainable.

Cerebral palsy (CP) is characterized by deficits in selective motor control, including abnormal posture, poor trunk stability, and impaired dynamic balance, all of which contribute to inadequate postural control and significant limitations in daily activities [7]. Motor [28] and sensory [29] impairments in children with CP negatively affect not only trunk balance but also the functional use of the upper extremities. Difficulties in reaching, extending, grasping, releasing, and object manipulation are commonly observed, which significantly impacts the quality of daily function [30]. Moreover, previous studies have highlighted a strong relationship between upper extremity function and trunk control [4].

While previous studies have examined the relationship between core stabilization and upper extremity function [4,31,32,33], only a few have systematically assessed the direct impact of core exercises on functional upper limb development in children with CP [15,21,22]. For instance, Abd-Elfattah et al. [15] (52 participants) and Akbaş & Günel [21] (36 participants) used the BBT, Jebsen Taylor Hand Function Test (JTHFT), and Quality of Upper Extremity Skills Test (QUEST) to evaluate upper limb function, focusing solely on unilateral dexterity and movement quality. However, these studies did not assess bimanual coordination, which plays a central role in functional independence during daily tasks. Similarly, Abd-Elhameed et al. [22] examined only reaching tasks without evaluating distal hand functions such as grasping or object manipulation. Although these studies reported improvements in isolated motor parameters (e.g., BBT and JTHFT scores), their clinical relevance remains limited, as most daily activities (e.g., opening bottles, buttoning clothes, using scissors) require coordinated bimanual function and postural stability.

In contrast, our randomized controlled trial simultaneously evaluated both unilateral and bimanual upper extremity performance within a single intervention framework. By incorporating the AHA, we quantitatively assessed how children with CP used their affected limb during purposeful bimanual tasks (e.g., assembling Lego blocks, opening containers, manipulating toys), thereby capturing functional challenges and compensatory strategies encountered in everyday life. A major strength of the AHA is its ecological validity; video recorded, play-based assessments reflect naturalistic behavior while minimizing the influence of artificial laboratory conditions. This methodological approach addresses a critical gap in the literature, as no previous randomized controlled trial, to our knowledge, has concurrently examined the effects of core stabilization on proximal stability (via TCMS and PBBS) and its indirect impact on bimanual hand use.

Sensorimotor impairments, deficits in sensorimotor integration, and challenges in motor planning frequently observed in children with cerebral palsy disrupt bimanual coordination [34]. Bimanual tasks are inherently more complex than unilateral ones, as they often involve coordinated actions such as reaching, grasping, lifting, and manipulating objects using both hands simultaneously [35,36]. In our study, the between-group comparison of difference scores revealed a statistically significant improvement in the BBT, which assesses unilateral hand use. However, no significant difference was found in AHA scores, which evaluate bimanual function. These findings suggest that while gains were observed in unilateral motor performance following the intervention, these improvements did not translate into enhanced bimanual coordination. This lack of intergroup difference may be attributed to several factors. First, bimanual hand use often requires complex bilateral coordination, which may not be sufficiently addressed by core stabilization exercises alone. Second, the relatively short intervention period (4 weeks) may have been insufficient to induce measurable changes in bimanual functional capacity, which typically improves over longer training durations. Third, the AHA, as a specialized measure of bimanual performance, might capture subtler aspects of hand function that are less responsive to general trunk-targeted interventions. Future studies should explore combining core stabilization with targeted upper limb interventions to enhance bimanual outcomes. Another plausible explanation for the absence of significant intergroup differences in bimanual skills is that both groups underwent comparable bimanual training as part of their occupational therapy programs. This finding suggests that standard rehabilitation alone may be sufficient to elicit improvements in bimanual function, whereas the added benefit of core stabilization exercises may become evident only in the context of more complex or prolonged functional tasks.

Children with CP commonly experience deficits in trunk control, dynamic balance, and coordination, which significantly limit their daily activities and reduce their quality of life [7]. Impaired trunk control has been shown to negatively impact the performance of gross motor skills that require balance, such as sitting, reaching, standing, and walking. It also affects oral motor functions like eating, swallowing, and speaking [37]. Trunk control and balance are therefore critical for functional independence in children with CP during daily activities. Adequate core stabilization in a seated position enhances overall functionality and encourages active participation in everyday tasks. Improved core stability enables children to use their arms and legs more effectively during activities [38,39]. For children with CP, developing core stability and trunk balance is essential for performing ADLs such as reaching forward, rotating the trunk, picking up objects from the floor while seated, maintaining an upright sitting posture, and dressing tasks like putting on shoes or socks [7].

The literature describes a variety of methods aimed at improving trunk balance in children with CP [40,41,42]. One such method is the use of core stabilization exercises. Research has shown that core stabilization programs activate trunk muscles by enhancing proprioceptive input and improving the somatosensory regulation of the vestibular system [18].

In our study, significant improvements favoring the intervention group were observed in both intergroup comparisons and the analysis of changes in PBBS scores, which were used to assess functional balance. Furthermore, analysis of static stability, dynamic reaching, and total scores on the TCMS revealed that the core stabilization exercise program led to notable gains in the intervention group compared with the control group. These findings align with a growing body of literature on the effects of core stabilization exercises in children with CP [16,17,18,19,20,21]. For instance, Shin et al. [20] reported that an 8-week core program significantly improved all TCMS sub-scores in children with CP. Similarly, Afifa Munaf et al. [19] found that core exercises enhanced trunk stability and were associated with increased PBBS scores. El-Shemy et al. [16], who evaluated trunk muscle endurance, observed clinically meaningful improvements in both endurance and gait parameters following an 8-week program. Notably, among these studies, only Akbaş and Günel [21] assessed both trunk parameters and unilateral upper extremity functions, reporting significant improvements in the intervention group.

The positive outcomes observed in our study may be attributed to the ability of core stabilization exercises to improve the strength and endurance of trunk-stabilizing muscles while simultaneously enhancing the interaction between neural control and the musculoskeletal system. These improvements in the intervention group are likely due to the targeted strengthening of the abdominal, back, shoulder, and pelvic muscles. Beyond muscular strengthening, it is hypothesized that other essential components of trunk stability—such as trunk position sense [43,44] and feedforward-driven postural adjustments [45]—were also supported by the core exercises, contributing to the overall functional gains observed.

The relationship between upper extremity functions and trunk balance has been proven by studies [4,31,32,33]. In our study, the correlation between upper extremity unilateral/bilateral functions and the PBBS and TCMS assessment criteria in children with CP was examined, and a positive relationship was found in all parameters except for the correlation between AHA and the static sitting balance subtest of TCMS. When examining current studies investigating the relationship between trunk balance and upper extremity functions in children with CP, it is seen that results similar to ours have been obtained. Yıldız et al. [4] reported a positive correlation between TCMS scores and upper extremity function. Similarly, Cornejo et al. [33] found a moderate correlation between upper limb performance (measured via the BBT) and static balance, emphasizing the role of trunk control in coordination and fine motor tasks. However, both studies focused exclusively on unilateral upper extremity function. In contrast, Darji and Divan [31] investigated bimanual upper extremity functions in 34 children with CP and identified a strong positive correlation with trunk balance. Likewise, Kim et al. [32] assessed both unilateral and bilateral functions and reported positive correlations between TCMS scores and performance on the BBT, ABILHAND-Kids, and QUEST. These findings underscore the importance of evaluating trunk control when assessing upper extremity function in children with CP.

While many studies have demonstrated the positive effects of core stabilization exercises on upper extremity functions, one study reported no significant advantage of these exercises over conventional physical therapy in improving upper limb performance [46]. In a randomized controlled trial involving stroke patients, El-Nashar et al. attributed the lack of significant results to the long disease duration (more than six months) and the short intervention period. They concluded that the treatment duration was insufficient to yield meaningful improvements in muscle strength or upper extremity function between groups. Similarly, a study by Chung et al. [47] investigated the effects of core stabilization exercises on dynamic balance and gait in stroke patients. Although the study group showed improved gait speed post-treatment, no significant differences were found in other outcome measures.

In our study, we aligned the core components of the intervention with the relevant F-words. These six F-words represent key areas to consider in the rehabilitation of children with developmental disabilities. Grounded in the ICF framework, they aim to translate theoretical concepts into practical, everyday strategies for children and their families [27]. Functioning, as defined by the ICF, refers to what a child does in their environment, encompassing the activities they perform and how they interact with their surroundings. In our study, activities such as balance training, gait training, standing on uneven surfaces, stair climbing, single- and double-leg jumping, dual-task exercises, goal-directed tasks, bimanual activities, grasping, reaching, releasing, and home exercise programs were categorized under this domain. Fitness relates to body function and structure, emphasizing the importance of physical activity, which often poses challenges for individuals with disabilities. In our intervention, neurodevelopmental treatments, stretching, strengthening, breathing, and weight-shifting exercises were included under this category. Fun refers to activities the child enjoys and the level of engagement they show. In our study, we incorporated toys during bimanual activities—such as Legos, puppets, animal imitations (e.g., donkey kicks), games with a large Pilates ball, and small ball throwing—to capture the child’s attention and boost motivation. Friends highlights the role of peer interaction and social connection. This was supported in our sessions when children played together, shared tasks (e.g., passing a ball), and interacted during dual or bimanual activities. Family represents the child’s home environment and emphasizes family-centered care. Therapists provided parents with strategies to integrate therapeutic activities into daily routines, ensuring continuity between sessions. Families were also actively involved in goal setting tailored to their children’s individual needs. However, all interventions were delivered in a clinical setting and not generalized to natural environments such as the home, school, or playground. This represents a limitation of our study. To address this, we provided home programs aligned with the family domain to partially bridge this gap.

Involving families more directly in the therapeutic process is essential for maximizing functional gains and ensuring long-term success. Caregiver-delivered home exercises not only improve carryover but also foster greater empowerment and engagement. Moreover, enhancements in motor function and independence may have significant psychosocial benefits, including increased self-confidence in children, reduced caregiver stress, and stronger parent–child interaction. Future interventions should integrate strategies to address both the functional and emotional dimensions of disability for a more holistic impact.

Future reflects a forward-looking perspective, focusing on short- and long-term goals. While this domain is central to the F-words framework, our study did not include long-term planning (e.g., adolescence or transition phases), which constitutes another limitation.

A review of the existing literature on the functional outcomes of core stabilization exercises in children with cerebral palsy (CP) reveals that most interventions span longer than four weeks [15,18,19,20,21,22]. In three studies specifically examining the impact of core exercises on upper extremity function, treatment durations were 8, 8, and 12 weeks, respectively, with all reporting significant improvements [15,21,22]. Although our study demonstrated significant gains within a 4-week period, the relatively short duration may have limited the extent of improvement, particularly in complex motor skills such as bimanual coordination. Future studies employing longer intervention periods may yield stronger or more sustained effects, contributing to a clearer understanding of the dose–response relationship in core stabilization programs.

While this study included children aged 5–12 years with both hemiplegic and diplegic CP, subgroup analyses based on age or CP subtype were not performed due to the limited sample size and the need to preserve statistical power. However, it is important to recognize that these variables may significantly influence treatment responsiveness. For example, younger children may benefit more due to greater neuroplasticity, while children with hemiplegic CP may respond differently than those with diplegic CP due to variations in motor impairment severity, unilateral versus bilateral involvement, and compensatory movement strategies.

Furthermore, age-related differences in cognitive function, attention span, and motivation could affect engagement with therapy, potentially influencing treatment adherence and outcomes. The interplay of biological and behavioral factors may either enhance or attenuate intervention effects across different subgroups. Future research should be adequately powered to conduct stratified or interaction analyses, enabling a more precise understanding of how age and CP subtype moderate treatment outcomes. Such insights would be essential for designing individualized, evidence-based rehabilitation protocols that optimize therapeutic results for specific patient populations.

These findings may extend beyond the domains of physiotherapy and occupational therapy, suggesting broader implications for multidisciplinary rehabilitation approaches. Core stabilization exercises could be effectively integrated into collaborative models involving speech language pathologists, psychologists, and special educators. These professionals may reinforce communication, behavioral regulation, and cognitive skills in tandem with motor improvements. Furthermore, embedding such interventions into everyday settings like schools, early childhood centers, or community-based programs could promote consistency, accessibility, and functional carryover, especially for children lacking regular access to hospital-based care.

Our study has several limitations. In our study, children with CP underwent a 4-week treatment. However, a review of the existing literature reveals that in previous studies, core stabilization exercises were applied to children with CP for durations longer than 4 weeks. This suggests that the duration of treatment in our study might have been insufficient to observe more substantial results. Another limitation of our study is the potential influence of age on functional outcomes. Although we included children aged 5–12 years to capture a developmental spectrum, age-related differences in motor learning, trunk control maturation, or compliance with exercises may have affected the results. For instance, younger children might respond differently to core stabilization training due to variations in neuromuscular development or attention span compared with older participants. While we statistically controlled for baseline age differences (median age: 5.5 vs. 7.5 years, *p* = 0.055), future studies could stratify analyses by age subgroups (e.g., 5–8 vs. 9–12 years) to explore these effects more rigorously. Additionally, longer intervention periods might mitigate age-related variability by allowing younger children more time to adapt. Another limitation is that the study was conducted only with children at GMFCS and MACS levels I, II, and III. Future studies should include children with CP from different levels of GMFCS and MACS. The fourth limitation of our study is that long-term outcomes were not evaluated. While our results demonstrate significant short-term improvements in balance and upper extremity function following core stabilization exercises, the sustainability of these gains remains unclear. Future studies should incorporate follow-up evaluations at 3, 6, or 12 months post-intervention to determine whether the observed benefits are maintained over time or require periodic reinforcement. Such data would clarify the need for booster sessions or home-based maintenance programs in clinical practice.

In addition to clinical considerations, the broader implementation of core stabilization exercises presents practical challenges. These include limited access to trained professionals, variability in service delivery across regions, and time constraints in school or home settings. Nonetheless, the emergence of telerehabilitation platforms and digital tools offers promising opportunities to enhance accessibility and adherence, particularly in underserved or remote communities. Incorporating video-based demonstrations, real-time remote monitoring, or caregiver-led protocols could support scalable and cost-effective delivery of these interventions.

Another limitation of this study is that multiple comparison adjustments were not performed, as each parameter was treated as an independent univariate outcome. This may increase the risk of type I error, and the results should be interpreted accordingly.

The exclusion of children with common CP comorbidities (e.g., epilepsy, intellectual disability) may substantially reduce the representativeness of our sample. Given that up to 52% of children with CP experience at least one comorbidity, our findings are primarily applicable to a subset of the CP population without these conditions. Future studies should prioritize inclusive recruitment strategies to evaluate intervention efficacy in more heterogeneous cohorts. Finally, the lack of examination of the impact of core stabilization exercises on the children’s quality of life and treatment satisfaction is a limitation of our study.

## 5. Conclusions

This study aimed to investigate the effects of core stabilization exercises, in addition to classical physiotherapy and rehabilitation methods, on unilateral and bimanual upper extremity functions and balance parameters in children with cerebral palsy. Our study is valuable as the first in the literature to specifically evaluate the effects of core stabilization exercises on both balance and upper extremity function using the Assisting Hand Assessment (AHA) to measure bimanual hand use. The findings suggest that core stabilization exercises significantly contribute to improvements in balance and unilateral hand function (as measured by the Box and Block Test) but do not result in significant improvements in bimanual function (AHA). In this context, emphasizing trunk control in physiotherapy and rehabilitation interventions is crucial for enhancing balance and unilateral motor performance. However, to improve bimanual coordination, additional or more targeted interventions may be necessary.

## Figures and Tables

**Table 1:** Comparison of intervention components between control and study groups.

Component	Control Group	Study Group
Physiotherapy program (ROM, strengthening, balance, gait training, etc.)	5 days/week, 45 min/session, 4 weeks	5 days/week, 45 min/session, 4 weeks
Occupational therapy (bimanual activities, fine/gross motor tasks)	5 days/week, 45 min/session, 4 weeks	5 days/week, 45 min/session, 4 weeks
Core stabilization exercises	✖ No	Additional ~45 min/day, supervised, 5 days/week, 4 weeks
Total daily therapy duration	~90 min	~135 min (combined physiotherapy + occupational therapy + core exercises)
Supervision and adherence monitoring	Trained physiotherapists, adherence > 90% (checklists)	Trained physiotherapists, adherence > 90% (video audits + checklists)

**Table 2:** Demographic and clinical characteristics of study and control groups, including age, gender, CP type, GMFCS levels, and MACS levels.

	Study Group (*n* = 18)	Control Group (*n* = 18)	*p*
**Age (years), median (IQR)**	5.5 (5.0–6.5)	7.5 (5.87–10.0)	0.055 ^a^
**Sex, n (%)**			1.0 ^b^
Girl	9 (50)	8 (44.4)	
Boy	9 (50)	10 (55.6)	
**CP type, n (%)**			0.443 ^b^
Hemiplegic type	6 (33.3)	3 (16.7)	
Diplegic type	12 (66.7)	15 (83.3)	
**GMFCS levels, n (%)**			0.695 ^b^
I	6 (33.3)	4 (22.2)	
II	6 (33.3)	5 (27.8)	
III	6 (33.3)	9 (50)	
**MACS levels, n (%)**			0.486 ^b^
I	8 (44.4)	8 (44.4)	
II	7 (38.9)	4 (22.2)	
III	3 (16.7)	6 (33.3)	

IQR: Interquartile range; n: Number of people; ^a^ Mann–Whitney U test; ^b^ Chi-square test.

**Table 3:** Correlation analysis of evaluation parameters.

	PBBS *	TCMS Static Sitting Balance *	TCMS Selective Movement Control *	TCMS Dynamic Reaching *	TCMS Total Score *
**BBT**	**r** 0.406	0.292	0.448	0.392	0.453
	***p*** **0.014**	0.084	**0.006**	**0.018**	**0.005**
**AHA**	**r** 0.410	0.260	0.425	0.364	0.418
	***p*** **0.013**	0.125	**0.010**	**0.029**	**0.011**

*: Spearman’s Rho correlation coefficient.

**Table 4:** Intragroup and intergroup comparison of BBT and AHA data.

	Study Group (*n* = 18)	Control Group (*n* = 18)	*p* ^a^
**BBT, median (IQR)**			
Before treatment	12.0 (8.5–17.2)	14.0 (7–26.7)	0.252
After treatment	18.50 (15.0–26.0)	15.0 (8.75–30.0)	0.767
*p* ^b^	**<0.001**	**<0.001**	
Cohen’s d	1.595	1.622	
**AHA** **, median (IQR)**			
Before treatment	54.0 (39.0–77.0)	61.5 (46.7–77.0)	0.673
After treatment	66.0 (50.5–80.0)	65 (53.5–78.5)	0.888
*p* ^b^	**0.001**	**0.001**	
Cohen’s d	1.317	1.255	

IQR: Interquartile range. ^a^ *p* value of between-group analyses (Mann–Whitney U test). ^b^ *p* value of within-group analyses. (Wilcoxon Signed Rank Test). Cohen’s d: Effect size.

**Table 5:** Intragroup and intergroup comparison of PBBS and TCMS data.

	Study Group (*n* = 18)	Control Group (*n* = 18)	*p* ^a^	Cohen’s d
**PBBS, median (IQR)**				
Before treatment	29.0 (10.5- 48.5)	11.5 (2.0–50.5)	0.181	0.467
After treatment	43.5 (19.7–52.7)	14.5 (3.5–51.2)	**0.011**	0.925
*p* ^b^	**<0.001**	**0.004**		
Cohen’s d	1.587	1.090		
**TCMS**				
***Static sitting balance, median (IQR)***				
Before treatment	14.0 (9.75–16.0)	12.0 (6.0–16.0)	0.323	0.345
After treatment	16.0 (14.0–20.0)	12.0 (8.0–16.0)	**0.003**	1.11
*p* ^b^	**<0.001**	**0.024**		
Cohen’s d	1.534	0.809		
***Selective movement control, median (IQR)***				
Before treatment	14.0 (8.5–17.0)	11.0 (2.25–23.0)	0.606	0.397
After treatment	20.0 (15.25–25.2)	13.0 (4.0–26.2)	0.171	1.09
*p* ^b^	**<0.001**	**0.007**		
Cohen’s d	0.929	0.645		
***Dynamic reaching, median (IQR)***				
Before treatment	10.0 (7.0–10.0)	9.5 (2.0–10.0)	0.308	0.175
After treatment	10.0 (10.0–10.0)	10.0 (3.50–10.0)	**0.037**	0.472
*p* ^b^	**0.012**	0.066		
Cohen’s d	1.588	1.000		
***Total Score, median (IQR)***				
Before treatment	35.0 (26.0–43.0)	32.5 (8.0–49.2)	0.673	0.148
After treatment	44.0 (39.7–53.2)	37.0 (14.0–51.5)	**0.044**	0.712
*p* ^b^	**<0.001**	**0.001**		
Cohen’s d	1.592	1.322		

IQR: Interquartile range. ^a^ *p*-value of between-group analyses (Mann–Whitney U test). ^b^ *p* value of within-group analyses. (Wilcoxon Signed Rank test). Cohen’s d: Effect size.

**Table 6:** Between-group comparison of difference scores.

	Study Group (*n* = 18)	Control Group (*n* = 18)	*p* ^a^	Cohen’s d
**BBT, median (IQR)**	7.0 (5.5–8.0)	1.5 (1.0–4.2)	**<0.001**	2.323
**AHA, median (IQR)**	6.0 (0.75–12.5)	2.5 (0.0–5.0)	0.051	0.7
**PBBS, median (IQR)**	8.0 (4.75–14.0)	1.0 (0.0–1.2)	**<0.001**	2.723
**TCMS**				
***Static sitting balance*** ***, median (IQR)***	3.5 (2.0–4.5)	0.0 (0.0–1.0)	**<0.001**	2.234
***Selective movement control*** ***, median (IQR)***	6.0 (3.0–9.2)	1.0 (0.0–4.0)	**0.001**	0.541
***Dynamic reaching*** ***, median (IQR)***	0.0 (0.0–3.0)	0.0 (0.0–0.2)	0.192	1.291
***Total score, median (IQR)***	12.0 (5.0–15.0)	2.0 (0.75–6.0)	**<0.001**	1.856

IQR: Interquartile range. ^a^ *p*-value of between-group analyses (Mann–Whitney U test). Cohen’s d: Effect size.

## Data Availability

The data presented in this study are available on request from the corresponding author. The data are not publicly available due to privacy and ethical restrictions.

## References

[1] Carlberg E.B., Bower E., Hadders Algra M., Brogren Carlberg E. (2008). Postural control in sitting children with CP: In Postural Control: A Key Issue in Developmental Disorders.

[2] Bax M., Goldstein M., Rosenbaum P., Leviton A., Paneth N. (2005). Proposed definition and classification of cerebral palsy. Dev. Med. Child Neurol..

[3] Rosenbaum P., Paneth N., Leviton A., Goldenstein M., Bax M. (2007). A report: The definition and classification of cerebral palsy, April 2006. Dev. Med. Child Neurol..

[4] Yildiz A., Yildiz R., Elbasan B. (2018). Trunk control in children with cerebral palsy and its association with upper extremity functions. J. Dev. Phys. Disabil..

[5] Willson J.D., Dougherty C.P., Ireland M.L., Davis I.M. (2005). Core stability and its relationship to lower extremity function and injury. J. Am. Acad. Orthop. Surg..

[6] Szopa A., Domagalska-Szopa M. (2024). Postural Stability in Children with Cerebral Palsy. J. Clin. Med..

[7] Heyrman L., Desloovere K., Molenaers G., Verheyden G., Klingels K., Monbaliu E., Feys H. (2013). Clinical characteristics of impaired trunk control in children with spastic cerebral palsy. Res. Dev. Disabil..

[8] Carlberg E.B., Hadders-Algra M. (2005). Postural dysfunction in children with cerebral palsy: Some implications for therapeutic guidance. Neural Plast..

[9] Van der Heide J.C., Hadders-Algra M. (2005). Postural muscle dyscoordination in children with cerebral palsy. Neural Plast..

[10] Arner M., Eliasson A.C., Nicklasson S., Sommerstein K., Hägglund G. (2008). Hand function in cerebral palsy. Report of 367 children in a population-based longitudinal health care programme. J. Hand Surg..

[11] Martinie O., Mercier C., Gordon A.M., Robert M.T. (2021). Upper limb motor planning in individuals with cerebral palsy aged between 3 and 21 years old: A systematic review. Brain Sci..

[12] Cooper J., Majnemer A., Rosenblatt B., Birnbaum R. (1995). The determination of sensory deficits in children with hemiplegic cerebral palsy. J. Child Neurol..

[13] Rose J., Wolff D.R., Jones V.K., Bloch D.A., Oehlert J.W., Gamble J.G. (2002). Postural balance in children with cerebral palsy. Dev. Med. Child Neurol..

[14] Liao H.F., Jeng S.F., Lai J.S., Cheng C.K., Hu M.H. (1997). The relation between standing balance and walking function in children with spastic diplegic cerebral palsy. Dev. Med. Child Neurol..

[15] Abd-Elfattah H.M., Aly S.M. (2021). Effect of core stability exercises on hand functions in children with hemiplegic cerebral palsy. Ann. Rehabil. Med..

[16] El Shemy S.A. (2018). Trunk endurance and gait changes after core stability training in children with hemiplegic cerebral palsy: A randomized controlled trial. J. Back Musculoskelet. rehabilitation.

[17] Ali M.S. (2019). Impact of core stability education on postural control in children with spastic cerebral palsy. Bull. Fac. Phys. Ther..

[18] El-Basatiny H.M.Y., Abdel-Aziem A.A. (2015). Effect of trunk exercises on trunk control, balance and mobility function in children with hemiparetic cerebral palsy. Int. J. Ther. Rehabil. Res..

[19] Munaf A., Mehboob S., Razzaq M., Younas M., Umair S., Waseem I., Gul S. (2022). Effect of trunk exercises on trunk control, balance, and mobility function in children with hemiparetic CP. Pak. J. Med. Health Sci..

[20] Shin J.W., Song G.B., Ko J. (2017). The effects of neck and trunk stabilization exercises on cerebral palsy children’s static and dynamic trunk balance: Case series. J. Phys. Ther. Sci..

[21] Akbas A.N., Gunel M.K. (2019). Effects of trunk training on trunk, upper and lower limb motor functions in children with spastic cerebral palsy: A stratified randomized controlled trial. Konuralp Med. J..

[22] Abd-Elhameed N.H., Kamal H.M., Abbass M.E. (2025). Effect of core stability exercises on upper limb reaching in children with spastic hemiparetic cerebral palsy: A randomized controlled trial. Bull. Fac. Phys. Ther..

[23] Krumlinde-Sundholm L., Holmefur M., Kottorp A., Eliasson A.C. (2007). The Assisting Hand Assessment: Current evidence of validity, reliability, and responsiveness to change. Dev. Med. Child Neurol..

[24] Mathiowetz V., Federman S., Wiemer D. (1985). Box and block test of manual dexterity: Norms for 6–19 year olds. Can. J. Occup. Ther..

[25] Erden A., Acar Arslan E., Dündar B., Topbaş M., Cavlak U. (2021). Reliability and validity of Turkish version of pediatric balance scale. Acta Neurol. Belg..

[26] Ozal C., Ari G., Gunel M.K. (2019). Inter–intra observer reliability and validity of the Turkish version of Trunk Control Measurement Scale in children with cerebral palsy. Acta Orthop. Traumatol. Turc..

[27] Rosenbaum P., Gorter J.W. (2012). The ‘F-words’ in childhood disability: I swear this is how we should think!. Child Care Health development.

[28] Yun G., Huang M., Cao J., Hu X. (2023). Selective motor control correlates with gross motor ability, functional balance and gait performance in ambulant children with bilateral spastic cerebral palsy. Gait Posture.

[29] Erkek S., Çekmece Ç. (2023). Investigation of the relationship between sensory-processing skills and motor functions in children with cerebral palsy. Children.

[30] Jaspers E., Desloovere K., Bruyninckx H., Klingels K., Molenaers G., Aertbelien E., Feys H. (2011). Three-dimensional upper limb movement characteristics in children with hemiplegic cerebral palsy and typically developing children. Res. Dev. Disabil..

[31] Darji P.P., Diwan S.J. (2023). Correlation between trunk control and upper extremity function in subjects with cerebral palsy. IP Indian J. Neurosci..

[32] Kim D.H., An D.H., Yoo W.G. (2018). The relationship between trunk control and upper limb function in children with cerebral palsy. Technol. Health Care.

[33] Cornejo M.I., Roldan A., Reina R. (2022). What Is the Relationship between Trunk Control Function and Arm Coordination in Adults with Severe-to-Moderate Quadriplegic Cerebral Palsy?. Int. J. Environ. Res. Public Health.

[34] Gordon A.M., Bleyenheuft Y., Steenbergen B. (2013). Pathophysiology of impaired hand function in children with unilateral cerebral palsy. Dev. Med. Child Neurol..

[35] Blinch J., Doan J.B., Gonzalez C.L. (2018). Complexity of movement preparation and the spatiotemporal coupling of bimanual reach-to-grasp movements. Exp. Brain Res..

[36] Klingels K., Demeyere I., Jaspers E., De Cock P., Molenaers G., Boyd R., Feys H. (2012). Upper limb impairments and their impact on activity measures in children with unilateral cerebral palsy. Eur. J. Paediatr. Neurol..

[37] Kavanagh J., Barrett R., Morrison S. (2006). The role of the neck and trunk in facilitating head stability during walking. Exp. Brain Res..

[38] Fallang B., Saugstad O.D., Hadders-Algra M. (2000). Goal directed reaching and postural control in supine position in healthy infants. Behav. Brain Res..

[39] Ambegaonkar J.P., Mettinger L.M., Caswell S.V., Burtt A., Cortes N. (2014). Relationships between core endurance, hip strength, and balance in collegiate female athletes. Int. J. Sports Phys. Ther..

[40] Pulay M.Á., Kornis K., Dörnyei G.B., Szabó É.F., Horváth M., Matiscsák A., Túri I. (2024). Feasibility of Using Pulsed Electromagnetic Field Therapy to Improve the Dynamic Postural Balance of Children with Cerebral Palsy: A Randomized, Sham-Controlled Pilot Study. J. Clin. Med..

[41] ŞŞimşşek T.T., Türkücüoğğlu B., Çokal N., Üstünbaşş G., ŞŞimşşek İ.E. (2011). The effects of Kinesio® taping on sitting posture, functional independence and gross motor function in children with cerebral palsy. Disabil. Rehabil..

[42] Matusiak-Wieczorek E., Dziankowska-Zaborszczyk E., Synder M., Borowski A. (2020). The influence of hippotherapy on the body posture in a sitting position among children with cerebral palsy. Int. J. Environ. Res. Public Health.

[43] Ebenbichler G., Oddsson L. (2001). Sensory-motor control of the lower back: Implications for rehabilitation. Med. Sci. Sports Exerc..

[44] Peterka R. (2002). Sensorimotor integration in human postüral control. J. Neurophysiol..

[45] Caronni A., Cavallari P. (2009). Anticipatory postüral adjustments stabilize the whole upper-limb prior to a gentle index finger tap. Exp. Brain Res..

[46] El-Nashar H., El-Wishy A., Helmy H., El-Rwainy R. (2019). Do core stability exercises improve upper limb function in chronic stroke patients?. Egypt. J. Neurol. Psychiatry Neurosurg..

[47] Chung E.J., Kim J.H., Lee B.H. (2013). The effects of core stabilization exercise on dynamic balance and gait function in stroke patients. J. Phys. Ther. Sci..

